# A Long-Term Comparative Study Between One Anastomosis Gastric Bypass and Sleeve Gastrectomy

**DOI:** 10.1007/s11605-022-05515-6

**Published:** 2022-11-14

**Authors:** Andreas Plamper, Philipp Lingohr, Jennifer Nadal, Jonel Trebicka, Maximilian J. Brol, Anna Woestemeier, Sophia M.-T. Schmitz, Patrick H. Alizai, Ulf P. Neumann, Tom F. Ulmer, Karl P. Rheinwalt

**Affiliations:** 1 Department for Bariatric, Metabolic and Plastic Surgery, St. Franziskus-Hospital, Cologne, Germany; 2 Department for General, Visceral, Thoracic and Vascular Surgery, University Hospital of Bonn, Bonn, Germany; 3 Institute for Medical Biometrics, Informatics and Epidemiology, University Hospital of Bonn, Bonn, Germany; 4 Department of Internal Medicine B, WW University Muenster, Muenster, Germany; 5 Clinic for General, Visceral and Transplantation Surgery, University Hospital RWTH Aachen, Aachen, Germany

**Keywords:** Bariatric surgery, Sleeve gastrectomy, Mini-gastric bypass, One anastomosis gastric bypass, Morbid obesity, Long-term results

## Abstract

**Background:**

One anastomosis gastric bypass (OAGB) has become increasingly accepted in bariatric surgery and meanwhile represents the third most common procedure worldwide. While it shows promising weight loss results and comorbidity resolution, questions about issues such as reflux or nutritional deficiencies (ND) persist in the long term. On the other hand, the most frequently performed sleeve gastrectomy (SG) has to accept growing criticism regarding long-term results and reflux issues. There is a particular lack of long-term comparative data for both procedures. This study presents our long-term experience.

**Methods:**

We evaluated OAGB and SG patients retrospectively comparing for weight loss and resolution of comorbidities as well as perioperative and long-term complications in a follow-up period of 5 years.

**Results:**

Nine hundred eleven OAGB and 241 SG were included in the study. OAGB had a shorter operation time and hospital stay. Overall complication rate did not differ in both groups. Ulcers were more frequent in OAGB (7.7% vs. 1.7%, *p* = 0.001), whereas insufficient weight loss (IWL)/weight regain (WR) proved to be more prevalent in SG (25.7% vs. 6.4%, *p* < 0.001). The same held true for reflux (17.8% vs. 8.3%, *p* < .001). On the other hand, ND were more common in OAGB (20.0% vs. 12.0%, *p* = 0.005). Revisional surgery was more often indicated after SG. Analysis by linear mixed model showed that OAGB achieved a lower BMI/higher loss of BMI. Improvement of T2DM (94.6% vs. 85.2%, *p* = 0.008) and sleep apnea (88.8% vs. 78.8%, *p* = 0.01) was superior in OAGB.

**Conclusions:**

OAGB had a superior effect on weight loss as well as improvement of T2DM and sleep apnea. Furthermore, long-term problems such as IWL/WR and reflux were more related to SG. On the other hand, a malabsorptive procedure such as OAGB showed a higher risk for ND. Our findings support the available data in the literature.

## Introduction

Sleeve gastrectomy (SG) has become the most frequent bariatric procedure for the treatment of morbid obesity worldwide.^1^ SG is technically not demanding and has demonstrated promising results at least for the first few years, contributing considerably to the popularity of this operation.^2,3^ However, the procedure recently faced growing criticism due to modest long-term weight loss and/or increasing frequency of postoperative reflux.^4–7^

Conversely, one anastomosis gastric bypass (OAGB) became an accepted standard procedure in bariatric surgery^8–11^ and now represents the third most commonly performed bariatric procedure worldwide.^1^ A growing number of studies reported very good and sustainable results for weight loss and resolution of comorbidities.^12,13^ Moreover, several studies have been conducted to compare OAGB to other standard procedures, such as SG and Roux-en-Y gastric bypass (RYGB).^12,14–16^ Our initial results favoring OAGB over SG in patients with super-obesity (BMI > 50 kg/m^2^) as well as in patients with type 2 diabetes mellitus (T2DM) in short-term follow-ups have been published previously.^17–19^ However, questions about issues such as (biliary) reflux or nutritional deficiencies (ND) persist in the long term after OAGB, and long-term data comparing these procedures remain rare.^12,20,21^ This study was conducted to present our own subsequent single-center experience after 5 years with OAGB in comparison to SG, including a focus on those previously outlined potential late complications.

## Methods

### Data Collection

Data on all bariatric patients that underwent surgery in our unit (1) have been collected prospectively since 2006 until now. Informed consent for pseudonymized data registration, data analysis, and publication was obtained from all patients.

### Patient Selection

All consecutive patients that either received OAGB or SG were identified, excluding redo/revisional surgery. Only patients that had at least 1 year of follow-up were included. The following parameters were documented at baseline (prior to the operation): age, gender, preoperative weight/body mass index (BMI), and the comorbidities hypertension, sleep apnea, T2DM, and osteoarthritis. Perioperative data included duration of the procedure, length of hospital stay, perioperative complications according to the classification by Clavien/Dindo,^22^ and 30-day mortality.

### Follow-Up

Follow-up data were acquired at least yearly according to national guideline recommendations, including actual weight and development of comorbidities, as well as potential late complications such as stenosis, reflux, insufficient weight loss (IWL)/weight regain (WR), or ND. ND was defined as either one or a combination of the following laboratory findings: iron deficiency, lack of vitamins (A, B_1_, B_6_, B_9_, or B_12_), hypoprotein-/hypoalbuminemia or markers of hypocalcemia (hyperparathyroidism, vitamin D deficiency).

### Primary and Secondary Endpoints

The primary endpoint was defined as weight loss, determined by % of total body weight loss (TBWL), % of excess weight loss (EWL), BMI, and loss of BMI (Δ BMI) up to 5 years after the intervention. The above-listed perioperative parameters and the evolution of comorbidities were defined as secondary endpoints. Our definition of remission or improvement of comorbidities that is adopted to the Buchwald criteria has been published previously^23,24^: hypertension (normal blood pressure values without any or with reduced medication), T2DM (HbA1 < 6.0%, FPG > 100 mg/dl without any special diet or medication/under control with reduced need for medication), sleep apnea (no further need for CPAP therapy or improved results in somnography), osteoarthritis (symptoms resolved or improved).

### Surgical Technique

Our techniques of OAGB and SG have been described previously.^17^ In brief, for OAGB, a long and narrow gastric pouch was created starting 3 cm below crow’s foot using a 30 Fr bougie starting with black and subsequently green, gold, and blue cartridges for completion of the gastric transection (Powered Echelon Flex, Ethicon Endo-Surgery, New Brunswick, USA). An antecolic 3.5–5-cm-long end-to-side-gastroenterostomy was performed by linear stapling (blue cartridge) after determining the biliopancreatic limb in length depending on the patient’s BMI: 200 cm for BMI < 50 kg/m^2^, 250 cm for BMI 50–60 kg/m^2^, 300 cm for BMI ≥ 60 kg/m^2^ (in cases however of elderly patients, preexisting tendency towards more frequent bowel movements or patients with rather lower absolute numbers in weight and height, we tended to choose limb lengths 50 cm shorter). For SG, the greater curvature was dissected 4–6 cm proximal to the pylorus until about 1 cm lateral to the angle of His. Gastric resection including the complete fundus was then performed against a 40 Fr bougie again with (from caudalad to cephalad) black, green, gold, and blue cartridges. In SG, the proximal staple line was routinely reinforced by buttress material or oversewing. An intraoperative leakage test was performed in all operations.

### Statistical Analysis

Data were analyzed using SPSS version 27 (IBM Corp., IBM SPSS Statistics, Chicago, IL). We described the baseline characteristics for the overall population and different technique attainment levels using mean values and standard deviations for continuous variables. The values of categorical variables were presented as frequency distributions with percentages.

To show differences between techniques, the unpaired *t*-test was used for continuous variables and Fisher’s exact test for categorical variables.

A linear mixed-effects model was used to quantify the follow-up data for BMI, BMI change, %TBWL, and %EWL within the patient population. The random effects model used here included follow-up time, surgical technique, and their interaction. *P* < 0.05 was considered significant.

## Results

A total of 1152 patients were enrolled in our study. Nine hundred eleven patients (694 female/217 male) received OAGB, and 241 patients (149 female/92 male) underwent SG.

Patients with SG had a higher preoperative weight and BMI. Moreover, the percentage of male patients was higher in this group (Table 1). Both groups were comparable for age and associated comorbidities with the exception of hypertension, which was more frequent in patients with SG.

**Table 1:** Patients’ baseline characteristics

	Total (*N* = 1152)	OAGB (*N* = 911)	SG (*N* = 241)	*p* value
Age (years)	42 ± 11	42 ± 11	44 ± 11	0.031
Female *N* (%): Male *N* (%)	843 (73.2) 309 (26.8)	694 (76.2) 217 (23.8)	149 (61.8) 82 (34.0)	< 0.001
Weight (kg)	148.04 ± 28.7	145.37 ± 26.1	158.13 ± 35.24	< 0.001
BMI (kg/m^2^)	51.65 ± 8.07	50.97 ± 7.31	54.21 ± 10.07	< 0.001
Comorbidities (*N*, %)				
Hypertension	740 (64.3%)	560 (61.5%)	180 (74.7%)	< 0.001
Sleep apnea	699 (60.7%)	552 (60.6%)	147 (61.0%)	0.941
Type 2 diabetes	417 (36.2%)	319 (35.0%)	98 (40.7%)	0.114
Osteoarthritis	1095 (95.1%)	868 (95.3%)	228 (94.6%)	0.617

Values are mean ± standard deviation, unless indicated otherwise

*OAGB* one anastomosis gastric bypass, *SG* sleeve gastrectomy, *BMI* body mass index

Table 2 demonstrates that the OAGB procedure could be carried out in a shorter period of time with a shorter hospital stay than in SG. All operations were carried out laparoscopically without conversion. One patient in the SG group died within 30 days after the operation due to multi-organ failure after staple line leakage (30-day mortality). The overall complication rate according to the Clavien-Dindo-classification did not differ in both groups. In particular, the occurrence of serious events (e.g., bleeding, stenosis) was similar except for a higher leakage rate in SG (Table 3). Ulcers were more frequent in OAGB compared to SG (7.7% vs. 1.7%, *p* = 0.001), whereas IWL/WR (25.7% vs. 6.4%, *p* < 0.001) and reflux (17.8% vs. 8.3%, *p* < 0.001) proved to be more prevalent in SG than in OAGB. On the other hand, ND were more common in patients with a bypass (20% vs. 12%). Among these 182 patients with OAGB that were diagnosed with ND, the mean BP length was 220 cm ± 38 cm, while the mean BP length was similar in patients without ND (221 cm ± 36 cm). When dividing all patients with OAGB into subgroups with a BP length < 220 cm (97 pts. with ND, 22.9% vs. 326 pts. w/o ND, 77.1%) and ≥ 220 (85 pts. with ND, 17.4% vs. 403 pts. w/o ND, 82.6%), the ND rate was significantly higher in the subgroup with shorter limb lengths (*p* = 0.046).

**Table 2:** Perioperative data, total operations *N* = 1152

	OAGB (*N* = 911)	SG (*N* = 241)	*p* value
Duration of procedure (min)	77 ± 19	94 ± 31	< 0.001
Duration of stay (days)	4 ± 3	7 ± 13	< 0.001
Postoperative complications, Clavien-Dindo (*n*, %)			
Grade I	89 (9.8%)	26 (10.8%)	0.090
Grade II	18 (2.0%)	6 (2.5%)	
Grade III	24 (2.6%)	11 (4.6%)	
Grade IV	15 (1.6%)	7 (2.9%)	
Grade V	1 (0.1%)	2 (0.8%)	

Values are mean ± standard deviation, unless indicated otherwise

*OAGB* one anastomosis gastric bypass, *SG* sleeve gastrectomy

**Table 3:** Postoperative complications

	Total	OAGB	SG	*p* value
Leakage	19	11 (1.2%)	8 (3.3%)	0.040
Hemorrhage	29	26 (2.8%)	3 (1.3%)	0.244
SSI	7	4 (0.4%)	3 (1.2%)	0.164
Stenosis	12	8 (0.9%)	4 (1.7%)	0.125
Ulcer	74	70 (7.7%)	4 (1.7%)	0.001
IWL/WR	120	58 (6.4%)	62 (25.7%)	< 0.001
Reflux	119	76 (8.3%)	43 (17.8%)	< 0.001
ND	211	182 (20.0%)	29 (12.0%)	0.005

Values are absolute numbers with percentage in parenthesis

*OAGB* one anastomosis gastric bypass, *SG* sleeve gastrectomy, *SSI* surgical site infection, *IWL* insufficient weight loss, *WR* weight regain, *ND* nutritional deficiencies

**Fig. 1:**
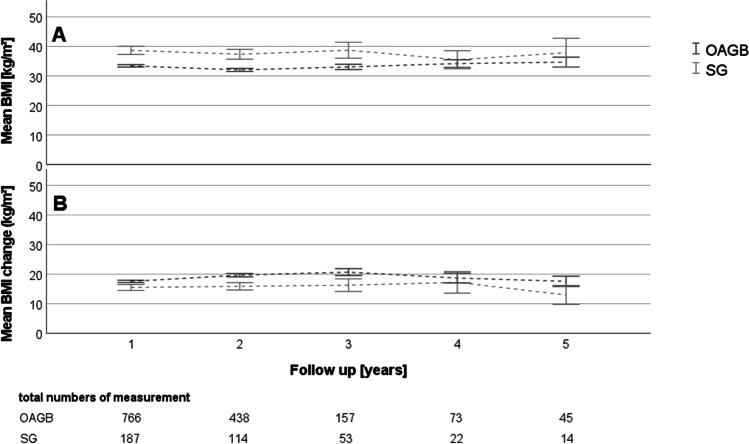
Mean values and their 95% confidence intervals for the measurements BMI (**A**) and BMI change (**B**) for the annual follow-ups

**Fig. 2:**
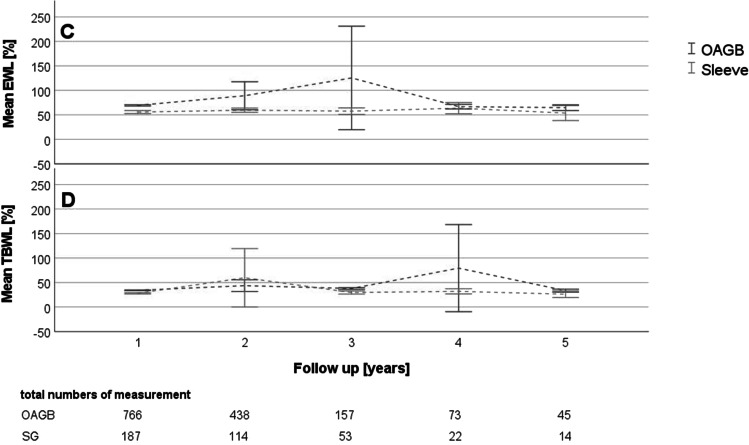
Mean values and their 95% confidence intervals for the measurements %EWL (**C**) and %TBWL (**D**) for the annual follow-ups

Applying the linear mixed model including the follow-up data of up to 5 years, it could be predicted that patients with OAGB were able to reach a lower BMI and a higher change in BMI, respectively (Table 4, Fig. 1). However, our model could not predict significant differences regarding %TBWL and %EWL between both procedures (Table 4, Fig. 2).

**Table 4:** Linear mixed model analysis of BMI, BMI change, TBWL, and EWL. The table contains coefficient estimates, standard error of coefficient estimates, and *p*-values obtained from likelihood ratio tests

	Parameter estimate	SE	*p*-value
BMI			
Intercept	38.07	0.53	0.0000
Visit per year	0.17	0.15	0.2450
OAGB	− 4.68	0.59	0.0000
SG	Reference		
OAGB * visit	− 0.43	0.17	0.0114
SG * visit	Reference		
BMI change			
Intercept	15.98	0.49	0.0000
Visit per year	− 0.20	0.14	0.1628
OAGB	1.51	0.55	0.0060
SG	Reference		
OAGB * visit	0.53	0.16	0.0012
SG * visit	Reference		
TBWL			
Intercept	34.92	12.83	0.0065
Visit per year	1.52	5.94	0.7980
OAGB	− 5.51	14.40	0.7022
SG	Reference		
OAGB * visit	4.23	6.74	0.53
SG * visit	Reference		
EWL			
Intercept	55.70	24.94	0.0256
Visit per year	0.94	11.55	0.9350
OAGB	11.48	28.00	0.6819
SG	Reference		
OAGB * visit	6.87	13.12	0.6007
SG * visit	Reference		

*BMI* body mass index, *OAGB* one anastomosis gastric bypass, *SG* sleeve gastrectomy, *TBWL* total body weight loss, *EWL* excess weight loss, *SE* standard error

**Fig. 3:**
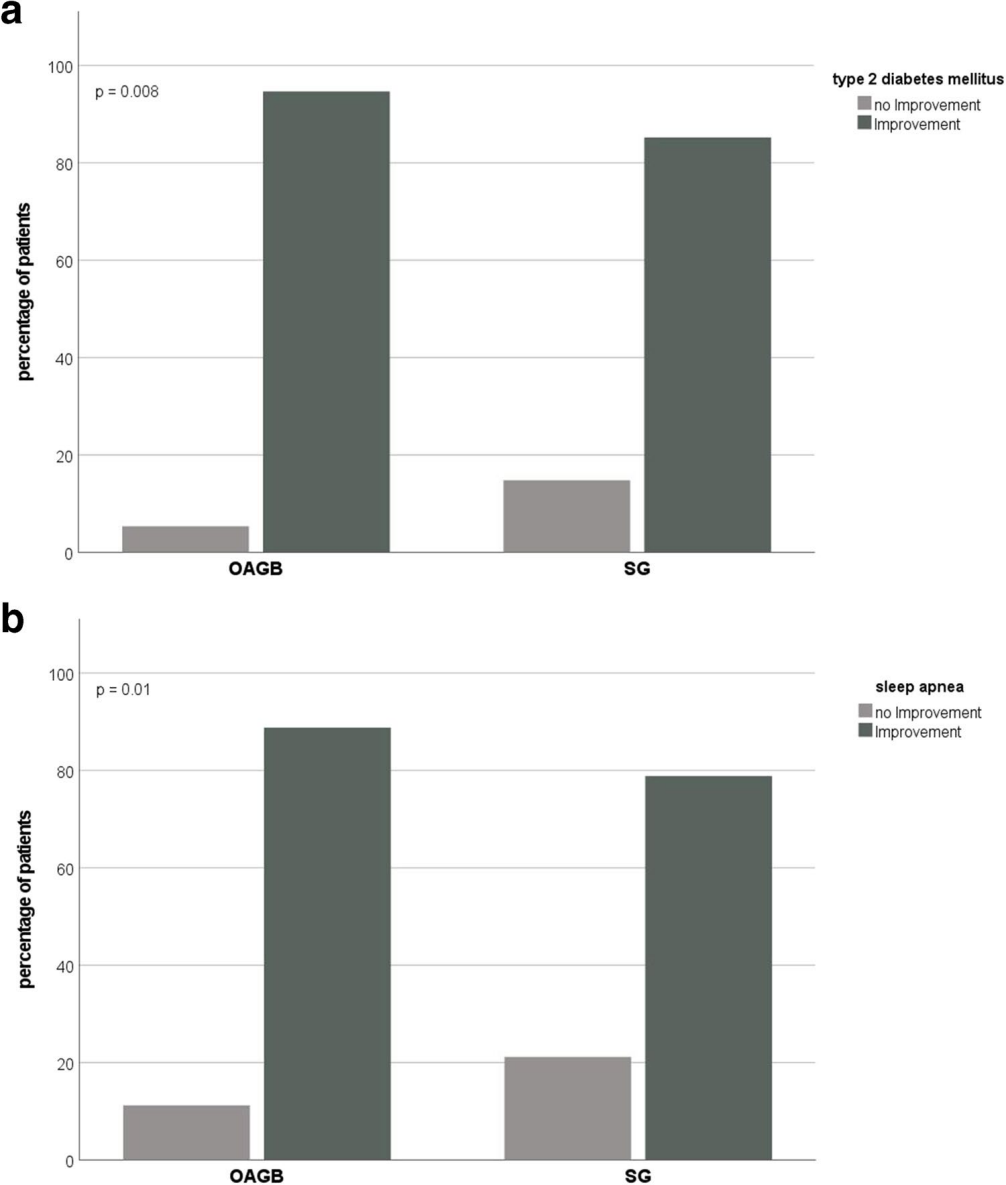
Evolution of preexisting comorbidities: Partial or total remission vs. no improvement or worsening, **a** type 2 diabetes mellitus, **b** sleep apnea (hypertension and osteoarthritis: data not shown)

Regarding the evolution of comorbidities, patients with OAGB showed a significantly better improvement of T2DM (94.6% vs. 85.2%, *p* = 0.008) and sleep apnea (88.8% vs. 78.8%, *p* = 0.01) than patients with SG (Fig. 3), whereas no differences were seen for hypertension (85.4% vs. 81.9%, *p* = 0.348) and osteoarthritis (82.0% vs. 76.6%, *p* = 0.128).

Mean follow-up was 25 months for OAGB and 32 months for SG. Additional follow-up data are shown in Table 5. While the follow-up rates were 85% for OAGB and 80% for SG after 1 year, they diminished over time towards 28.5% for OAGB and 17.1% for SG after 5 years. Six patients with SG died of causes unrelated to the operation in the course of time; none in the OAGB group.

**Table 5:** Follow-up data

	Years after the operation	1	2	3	4	5
Total	Follow-up attended	953	552	210	95	59
	Follow-up rate (%)	83.4	69.3	40.9	26.8	24.6
	Deceased (not eligible)	4	4	5	6	6
	Reoperated (not eligible)	8	26	39	45	48
	Lost to follow-up	183	244	303	260	181
OAGB	Follow-up attended	766	438	157	73	45
	Follow-up rate (%)	85.0	72.0	41.0	31.8	28.5
	Deceased (not eligible)	0	0	0	0	0
	Reoperated (not eligible)	6	18	20	15	14
	Lost to follow-up	135	170	226	157	113
SG	Follow-up attended	187	114	53	22	14
	Follow-up rate (%)	79.6	60.6	40.8	21.4	17.1
	Deceased (not eligible)	4	4	5	6	6
	Reoperated (not eligible)	2	8	19	30	34
	Lost to follow-up	48	74	77	103	68

*OAGB* one anastomosis gastric bypass, *SG* sleeve gastrectomy

The total reoperation rate was 8.1% after OAGB and 24.2% after SG. The most common indications for revisional surgery were related to weight loss (IWL/WR: 2.0% in OAGB and 18.6% in SG) and/or reflux issues (2.6% in OAGB and 16.5% in SG). Our criteria for eligibility for revisional surgery have been published previously: IWL and WR were thereby defined according to Reinhold’s criteria (BMI ≥ 35m^2^ and/or %EWL < 50%), while complicated reflux disease was defined as (acid or biliary or combined) reflux symptoms non-responsive to medical treatment and/or endoscopically proven esophagitis.^24,25^ Two patients with a bypass (0.2%) required shortening of the biliopancreatic (BP) limb because of persisting ND and one patient specifically demanded a complete reversal of the bypass situation.

## Discussion

We were previously able to demonstrate superior short-term results for OAGB concerning weight loss and T2DM resolution over SG.^17,18^ This subsequent study confirmed these initial findings by demonstrating better weight loss outcomes in terms of change in BMI/achieved BMI after OAGB when compared to SG after up to 5 years of follow-up. Furthermore, OAGB patients showed a higher resolution of T2DM and sleep apnea. Studies analyzing the long-term follow-up after OAGB and SG are scarce. In patients from South Asia, Jain et al.^26^ previously showed a higher effect with respect to weight loss, resolution of T2DM/hypertension, and improvement in quality of life in patients that underwent OAGB compared to SG after 5 years. A double-blind randomized trial by Lee et al.^27^ gave similar results with better metabolic effects for OAGB after 5 years; however, in this study, only patients with mild obesity were included (i.e., BMI < 35 kg/m^2^). Interestingly, other comparative long-term data (i.e., > 5 years) did not show a difference in T2DM resolution. Spanish researchers found lower BMI and higher excess BMI loss (EBMIL) in patients with T2DM and obesity for OAGB compared to SG and RYGB during a 5-year follow-up period but could not detect a difference in T2DM remission.^21^ In a recent publication from Taiwan, better TWL in patients with super-obesity could be demonstrated after OAGB when compared to SG and RYGB after a follow-up of up to 10 years while T2DM remission again did not differ between the groups.^28^ In regard to weight loss, we also recently published superior %TBWL and %BMI loss results for OAGB over SG in patients with super-super-obesity.^19^ A systematic review from 2017 detected only 3 additional studies reporting on better %EWL after 5 years for OAGB as well as higher remission rates for T2DM and sleep apnea.^12^

In our study, the OAGB procedure was shorter in duration than SG. The findings in the literature seem to be inconsistent on this issue. While the absence of a gastrointestinal anastomosis in SG would suggest a shorter operation time, a review of the available literature led to the conclusion that both procedures are comparably long.^14,29^ One explanation could be that in the majority of non-randomized studies, SG was favored in difficult cases such as higher BMI, unfavorable visceral fat distribution type especially in male patients, or previous surgery with expected adhesions. This selection bias affected our study as well. Our results regarding shorter hospital stay after OAGB are nonetheless in agreement with systematic reviews.^12,29^ In our opinion, this could possibly be explained by better oral liquid tolerance in patients with OAGB due to the wide gastrojejunostomy.

Our analysis showed comparably low overall complication rates for both procedures and is concordant with the literature.^2,12^ In large reviews, OAGB was also regarded as a safe procedure with a low complication rate even in patients with a high BMI.^13,14^ The relatively higher leakage rate in our SG group might be explained by the decision to offer SG in more difficult cases when a bypass procedure was expected to be too challenging (e.g., previous abdominal surgery with expected distinct adhesions). Nevertheless, a leakage rate of 3.3% still represents an acceptable (low) rate after SG, compared to the literature.^30^

We have already stressed the point that long-term comparative outcomes of OAGB with SG are still considerably lacking.^12^ We now demonstrated a higher IWL/WR rate for SG in the long-term (overall rate 25.7%/indication for reoperation in 18.6% vs. 6.4%/2.0% for OAGB) supporting the growing skepticism against SG about the sustainability of the achieved weight loss and the incidence of (new-onset) reflux.^5,6,31,32^ Yet, its popularity is still on the rise because of its relative simplicity and its initially promising weight loss results.^2,7,32,33^ This discrepancy over time is well summarized in a meta-analysis by Guan et al.^34^: while the overall revision rate after SG of all studies included in this review was 10.4%, this rate increased with the duration of follow-up and accumulated to 22.6% in studies with a follow-up ≥ 10 years and underlines the importance of long-term data.

In our study, the total (acid, biliary, or combined) reflux rate over the observed time period amounted to 8.3% and proved to be significantly lower than in our SG group (17.8%). It is notable that the incidence of patients requiring surgery for conservatively untreatable reflux was even lower after OAGB (2.6%) compared to SG (16.5%). These findings are in accordance with other studies. In a recent RCT, Musella et al.^35^ demonstrated a higher risk/worsening of esophagitis due to increased acid exposure in the first year after SG in comparison to OAGB. Felsenreich and colleagues^4,31^ have found a conversion rate to RYGB of 14% due to intractable reflux after SG with a total rate of reflux of 38%; the same group showed that 59% suffered from WR 10 years after SG.

We have previously published a total reflux rate of 3.55% and a rate of ND of 4.19% after up to 3 years of follow-up, which were not increased compared to RYGB.^36^ The relevance of sole bile reflux in OAGB is still a matter of ongoing discussion, but the condition is also difficult to detect: studies applying multichannel impedance pH-metry or bile reflux scintigraphy did not show an increased but rather a decreased incidence of bile/alkaline esophageal reflux after the operation.^6,37^ Large OAGB series describe a reflux rate between 0.2 and 1.2% after up to 9 years of follow-up.^38–42^ We want to emphasize again that the reflux rate given in our analysis comprises acid, biliary, and combined reflux. We are aware that presently two case reports of esophagogastric junctional cancer exist so far.^43,44^ Taking into account that a potential malignancy might still occur decades after exposure to acid/alkaline reflux, we strongly support the current IFSO recommendations on regular endoscopic follow-up after bariatric procedures that might have an elevated risk for chronic reflux.^45^

Finally, the total ND rate was higher in the OAGB group (20% vs. 12%) and stresses the malabsorptive effect of this procedure. It is, however, likewise notable that only 2 patients with OAGB (0.2%) needed surgical revision because of conservatively non-treatable ND. This condition remains a point of discussion in the literature. The French YOMEGA-RCT reported an elevated risk for ND following OAGB with a 200 cm BP limb after 2 years of follow-up.^20^ In our study, however, we could not detect a higher rate of ND in OAGB with longer limb lengths. In conclusion, many authors including us recommended collecting more long-term data on weight development, reflux, and ND. Hopefully, our results help to answer some of these important questions.

The following study limitations must be admitted: first, only retrospective data were available (however prospectively collected). As already pointed out, a certain selection bias regarding the choice of procedure is undeniable. Second, the decreasing follow-up rates over time represent a typical problem in our national health system.

In conclusion, we were nevertheless able to analyze a sufficient number of patient-specific slopes and hereby support the available comparative data on OAGB and SG that demonstrate favorable treatment results and lower reoperation rates for OAGB after a period of up to 5 years. Conversely, OAGB showed more problems with malnutrition. While the ideal operation in bariatric surgery does not exist thus far, the long-term care of obese patients remains challenging. Close and subsequent long-term follow-up is of utmost importance to uncover problems over the long haul. The lack of randomized controlled data comparing OAGB to SG and other procedures remains an additional problem that should be targeted.
